# Characteristics of patients referred to Canary Island pneumology outpatient services for chronic obstructive pulmonary disease: the EPOCan study

**DOI:** 10.1186/s13104-022-05930-7

**Published:** 2022-02-10

**Authors:** Juan Marco Figueira-Gonçalves, José María Hernández-Pérez, Carlos Cabrera-López, Aurelio Luis Wangüemert-Pérez, Ignacio García-Talavera, Yolanda Ramallo-Fariña, Carolina Ramos-Izquierdo, Luis Manuel González-García, Sara Guanche-Dorta

**Affiliations:** 1 Pneumology and Thoracic Surgery Service, Unit for Patients with Highly Complex COPD, University Hospital Nuestra Señora de Candelaria, Santa Cruz de Tenerife, Spain; 2 University Institute of Tropical Disease and Public Health of the Canary Islands, University of La Laguna, Santa Cruz de Tenerife, Spain; 3 Pneumology Service, University Hospital Dr. Negrín, Gran Canaria, Spain; 4 Pneumology Service, San Juan de Dios Hospital, Tenerife, Spain; 5 Foundation of the Canary Islands Health Research Institute (FIISC), Santa Cruz de Tenerife, SpainHealth Services Research on Chronic Patients Network (REDISSEC), Madrid, Spain; 6 Primary Care Centre of the Canary Islands Public Health Service, Breña Baja, La Palma, Santa Cruz de Tenerife, Spain

**Keywords:** Chronic obstructive pulmonary disease, COPD, Cardiovascular, Morbidity

## Abstract

**Objective:**

Assessing patients with chronic obstructive pulmonary disease (COPD) accounts for 30% of all pneumology outpatient evaluations. COPD is a heterogeneous disease and generates a massive public health problem. Overall morbidity, particularly cardiovascular disease, challenges patient management. This is an observational, multicentre study, performed at four hospitals in the Canary Islands (Spain), aimed at characterising patients with COPD referred to pneumology outpatient services. Demographic variables, lung function, and morbidity were assessed.

**Results:**

Of the 877 included patients, 44.9% were active smokers with a mean (± SD) age of 68.2 ± 10.3 years. The median (IQR) score for the Charlson comorbidity index was 2 (2), and 70.6% of the patients were assigned high risk according to the Spanish Guidelines for COPD (GesEPOC) 2021. The degree of airflow obstruction defined by the GOLD 2021 stages 1, 2, 3, and 4 corresponded to 13.6%, 49%, 31%, and 6.3% of patients, respectively. The most frequently associated morbidities were arterial hypertension (59.5%), dyslipidaemia (54.3%), and type 2 diabetes mellitus (31.2%); 32% of the patients suffered heart disease. There is a high prevalence of active smoking, type 2 diabetes mellitus, and heart disease in patients referred for COPD to Canary Island pneumology outpatient services.

**Supplementary Information:**

The online version contains supplementary material available at 10.1186/s13104-022-05930-7.

## Introduction

Chronic obstructive pulmonary disease (COPD) constitutes a public health problem with a huge impact on socio-economic costs [1–3]. The disease is one of the most frequent reasons to seek medical care and accounts for 10% of primary care and 30% of respiratory outpatient attention [1]. Moreover, COPD is a complex and heterogeneous disease, characterised by chronic, barely reversible airflow limitation, mainly—at least in high human development index countries—on account of tobacco smoke [4].

A range of studies has focused on the high morbidity in these patients, which worsens their prognosis and results in additional challenges in their management [5–9]. The cardiovascular morbidity rate of patients with COPD in the Canary Islands seems to exceed the national level [10, 11]. To corroborate this observation, we performed a multicentre study assessing characteristics of patients with COPD who had been referred to pneumology outpatient services in the Canary archipelago.

## Main text

### Methods

#### Study design

An observational, cross-sectional, multicentre study was performed involving four historical cohorts of outpatients with COPD from the University Hospital Nuestra Señora de Candelaria (Tenerife), the Hospital San Juan de Dios (Tenerife), the University Hospital Dr. Negrín (Gran Canaria), and La Palma General Hospital (La Palma). The retrieved data covers a period from 2011 to 2020.

#### Study population

The study included 877 COPD patients where the following inclusion criteria were applied: (1) patient attending follow up in an outpatient pneumology service; (2) age > 40 years; (3) active or former smoker with a pack-year index (PYI) ≥ 10 or exposition to another known risk factor like smoke from home cooking and heating fuels, occupational dust or other chemicals; (4) a forced expiratory volume in 1 s (FEV_1_)/forced vital capacity (FVC) ratio < 70% upon administration of 400 μg of salbutamol. The presence of chronic respiratory diseases other than COPD, e.g., interstitial lung disease or pneumoconiosis, was considered an exclusion criterion, except historial of asthma.

#### Ethical approval

Clinical data extraction from electronic, medical records was authorised by the corresponding ethics committees (Ethics Committee for Clinical Research of the University Hospital Nuestra Señora de Candelaria, registry number CHUNSC_2021_41). Data were de-identified for analysis. In this study, informed consent was waived for its retrospective, non-interventional design and the use of anonymous clinical data.

### Variables

Variables included in the analyses were age, gender, body mass index (BMI [kg/m^2^]), history of tobacco consumption assessed as PYI, peripheral oxygen saturation by means of pulse oximetry (SpO2)—performed with the patient at rest in a sitting position—dyspnoea assessment using the modified Medical Research Council scale (mMRC scale), long-term home oxygen therapy and/or bi-level positive airway pressure (BiPAP) or continuous positive airway pressure ventilation (CPAP), the presence of chronic mucus hypersecretion, and the number of severe exacerbations requiring hospital stays during the year prior to the first visit as an outpatient. Chronic mucus hypersecretion was registered in all the corresponding databases. Previous admissions were confirmed reviewing hospital records. Data on the associated morbidities arterial hypertension (AHT), type 2 diabetes mellitus (T2DM), dyslipidaemia (DLP), obesity (defined as BMI ≥ 30 kg/m^2^), underweight (BMI < 18.5 kg/m^2^), atrial fibrillation (AF), ischaemic heart disease (IHD), chronic heart failure (CHF), cerebrovascular accidents (CVA), neoplasia (solid tumours, lymphoma, leukaemia), osteoporosis, and mood disorders (anxiety and depression) were obtained. Each associated morbidity was confirmed by a comprehensive review of the electronic, medical records, data from diagnostic procedures, and disease-specific therapies. The Charlson comorbidity index (CCI) not age-adjusted score [12] and the BODEx index were determined for each patient. Forced spirometry data following bronchodilation was recorded as FEV_1_%, FVC%, and the FEV_1_/FVC ratio. The patients' degree of airflow obstruction was classified according to the Global Initiative for Chronic Obstructive Lung Disease (GOLD) document 2021 [4]. Based on the Spanish guidelines GesEPOC 2021, patients were also classified as low risk vs high risk patients [13].

### Statistical analysis

In agreement with published work [14], the representative sample size for the population with COPD in the Canary Islands was 599. This number was calculated taking into account the approximately 240,000 persons with COPD in the Canary Islands and the total of 2,500,000 inhabitants. An estimation error of 4%, a significance level of 5%, and a power of 80% were assumed. In addition, a maximum probability of 50% was considered to meet the sample size requirements for the different features of interest.

Qualitative variables were summarised as frequencies and percentages and continuous variables as means and standard deviations or median and interquartile range according to the normality of their distribution. For continuous normal variables, bivariate comparisons between independent samples were made using Student's t test. The Mann Whitney U test was used for continuous non- normal variables. Qualitative variables were tested by means of Chi-square or Fisher's exact test, as applicable. A p-value of < 0.05 was considered statistically significant. Analyses were performed using SPSS v.21 software.

## Results

The baseline characteristics of the 877 patients-comprising study population are given in Table 1. The patients' mean (± SD) age was 68.2 ± 10.3 years, 44.9% were active smokers and 20.1% of the population were women. The baseline characteristics of the patients according smoking status are given in Additional file 1: Table S1. The GOLD document 2021 stages 1, 2, 3, and 4, which define the degree of airflow obstruction, corresponded to 13.6, 49, 31, and 6.3% of the patients, respectively. The mean (± SD) % FEV_1_ was 57.9% (± 19.45), the median CCI was 2 (IQR = 2), and 70.6% were considered high risk patients according to GesEPOC 2021. The most frequently associated morbidities were AHT (59.5%), DLP (54.3%), and T2DM (31.2%).

**Table 1:** Baseline characteristics of patients with chronic obstructive pulmonary disease according to lung function and degree of dyspnoea

	Study population (n = 877)	FEV_1_ < 50% (n = 328; 37.4%)	FEV_1_ ≥ 50% (n = 549; 62.6%)	p-value	mMRC < 2 (n = 432; 49.3%)	mMRC ≥ 2 (n = 445; 50.7%)	p-value
Clinical data							
Mean age, years (SD)	68.2 (10.4)	69 (10.1)	67.7 (10.5)	0.12	66.4 (10.2)	69.9 (10.3)	** < 0.001**
Female, n (%)	176 (20.1)	70 (21.3)	106 (19.3)	0.467	90 (20.8)	86 (19.3)	0.577
Mean pack-year index (SD)	47.4 (27.5)	51.08 (26.7)	45.2 (27.8)	** < 0.001**	46.2 (26.8)	48.6 (28.2)	0.187
Active smoker, n (%)	394 (44.9)	140 (42.7)	254 (46.3)	0.584	186 (43.1)	208 (46.7)	0.240
Mean dyspnoea, mMRC score (SD)	1.6 (0.82)	1.9 (0.89)	1.4 (0.71)	** < 0.001**	0.87 (0.34)	2.3 (0.52)	** < 0.001**
mMRC score ≥ 2, n (%)	445 (50.7%)	221 (67.4)	224 (40.8)	** < 0.001**	–	–	**–**
Mean BMI (SD)	27.3 (5.3)	27.1 (5.7)	27.5 (5.09)	0.109	26.8 (5.1)	27.8 (5.5)	**0.01**
Median severe exacerbations in the previous year (IQR)	0 (0)	0 (0)	0 (0)	** < 0.001**	1 (1)	2 (2)	**0.001**
≥ 1 severe exacerbations in the previous year, n (%)	147 (16.8)	76 (23.2)	71 (12.9)	** < 0.001**	54 (12.5)	93 (20.9)	**0.001**
Median BODEx index (IQR)	2 (2)	3 (2)	1 (2)	** < 0.001**	1 (2)	3 (2)	** < 0.001**
BODEx > 4, n (%)	80 (9.1)	77 (23.5)	3 (0.5)	** < 0.001**	6 (1.74)	74 (16.6)	** < 0.001**
Mucus hypersecretion, n (%)	394 (44.9)	166 (50.6)	228 (41.5)	**0.009**	173 (40)	221 (49.7)	**0.004**
Long-term home oxygen therapy, n (%)	133 (15.2)	91 (27.7)	42 (7.7)	** < 0.001**	38 (8.8)	95 (21.3)	** < 0.001**
BiPAP or CPAP, n (%)	62 (8.4)	38 (14)	24 (5.1)	** < 0.001**	19 (5.4)	43 (11.1)	**0.006**
Mean no. diseases (SD)	2.61 (1.75)	2.58 (1.70)	2.63 (1.79)	0.98	2.25 (1.59)	2.95 (1.83)	** < 0.001**
Functional parameters							
FEV_1_/FVC	56.16 (11.0)	48.88 (11.5)	60.5 (8.1)	** < 0.001**	58.42 (9.7)	53.97 (11.8)	** < 0.001**
FEV_1_ (%)	57.9 (19.5)	38.36 (8.4)	69.6 (14.1)	** < 0.001**	63.52 (18.6)	52.44 (18.7)	** < 0.001**
FVC (%)	80.45 (21.89)	66.76 (18.75)	88.62 (19.43)	** < 0.001**	85.3 (21.7)	75.8 (21.1)	** < 0.001**
FEV_1_ ≥ 50%, n (%)	549 (62.6%)	–	–		325 (75.2)	224 (50.3)	** < 0.001**
Mean baseline SpO2, % (SD)	95.3 (2.1)	94.5 (2.4)	95.7 (1.6)	** < 0.001**	95.8 (1.8)	94.82 (2.2)	** < 0.001**
Morbidities, n (%)							
Arterial hypertension	522 (59.5)	197 (60.1)	325 (59.2)	0.801	234 (54.2)	288 (64.7)	**0.001**
Type 2 diabetes mellitus	274 (31.2)	106 (32.3)	168 (30.6)	0.596	100 (23.1)	174 (39.1)	** < 0.001**
Dyslipidemia	476 (54.3)	167 (50.9)	309 (56.3)	0.122	222 (51.4)	254 (57.1)	0.091
Obesity	239 (27.3)	88 (26.8)	151 (27.5)	0.073	99 (22.9)	140 (31.5)	**0.037**
Underweight	33 (3.8)	16 (4.9)	17 (3.1)	0.073	19 (4.4)	14 (3.1)	**0.037**
Ischaemic heart disease	151 (17.2)	46 (14)	105 (19.1)	0.053	52 (12)	99 (22.2)	** < 0.001**
Heart failure	94 (10.7)	40 (12.2)	54 (9.8)	0.275	34 (7.9)	60 (13.5)	**0.007**
Atrial fibrillation	152 (17.3)	54 (16.5)	98 (17.9)	0.599	54 (12.5)	98 (22)	** < 0.001**
Heart disease	281 (32)	101 (30.8)	180 (32.8)	0.54	104 (24.1)	177 (39.8	** < 0.001**
Cerebrovascular accident	71 (8.1)	28 (8.5)	43 (7.8)	0.711	29 (6.7)	42 (9.4)	0.139
Mood disorder	57 (6.5)	24 (7.3)	33 (6)	0.448	29 (6.7)	28 (6.3)	0.800
Osteoporosis	17 (1.9)	10 (3)	7 (1.3)	0.065	8 (1.9)	9 (2)	0.855
Bronchial asthma	111 (12.7)	48 (14.6)	63 (11.5)	0.173	56 (13)	55 (12.4)	0.788
Neoplasia	126 (14.4)	41 (12.5)	85 (15.5)	0.223	56 (13)	70 (15.7)	0.243
CCI ≥ 3	292 (33.3)	105 (32.1)	187 (34.1)	0.533	106 (24.5)	186 (41.9)	** < 0.001**
CCI, median (IQR)	2 (2)	2 (2)	2 (2)	0.545	1 (1)	2 (2)	** < 0.001**

Bold indicates p value < 0.05

*BMI* body mass index; *FEV*_*1*_ forced expiratory volume in 1 s; *FVC* forced vital capacity; *CCI* Charlson comorbidity index score, not age-adjusted; *mMRC* modified Medical Research Council scale; *SpO2* peripheral oxygen saturation by means of pulse oximetry measured with the patient at rest in a sitting position; *BiPAP* bi-level positive airway pressure; *CPAP* continuous positive airway pressure; *IQR* interquartile range

### Patient characteristics by sub-groups

#### According to lung function

Compared to patients with mild to moderate airflow obstruction, patients with FEV_1_ < 50% had a higher smoking load (PYI 45.2 ± 27.7 vs 51.1 ± 26.7; p < 0.001) and higher percentage of patients that required hospital admission in the previous year (12.9% vs 23.2%; p < 0.001). As to associated morbidities, no significant difference was detected (Table 1).

#### According to degree of dyspnoea

More aged individuals were detected in patients with mMRC ≥ 2 than in patients with a lower degree of dyspnoea (69.9 ± 10.3 vs 66.4 ± 10.2; p < 0.001). Table 1 details that the former also had a higher percentage of patients that required hospital admission in the previous year (20.9% vs 12.5%; p < 0.001), higher CCI scores (median 2 (IQR = 2) vs 1 (IQR = 1); p < 0.001), and a higher prevalence of cardiovascular morbidities.

#### According to history of exacerbations requiring hospital stays

Patients with hospital stays for exacerbation prior to attending outpatient services were older (70.1 ± 11.03 years vs 67.8 ± 10.22 years; p < 0.001) compared to individuals without hospital stays, had a higher percentage of patients with CCI score ≥ 3 (45.6 vs 30.9%; p < 0.001), and a higher prevalence of cardiovascular morbidities and conditions related to mood disorders (Table 2).

**Table 2:** Baseline characteristics of patients with chronic obstructive pulmonary disease according to previous hospital stays for exacerbation

	Hospital stay in the previous year		
	No (n = 730; 83.2%)	Yes (n = 147; 16.8%)	p-value
Clinical data			
Mean age, years (SD)	67.8 (10.2)	70.2 (11.0)	**0.008**
Female, n (%)	148 (20.3)	28 (19)	0.735
Mean pack-year index (SD)	47.3 (26.6)	47.9 (31.8)	0.802
Active smoker, n (%)	339 (46.4)	55 (37.4)	0.072
Mean dyspnoea, mMRC score (SD)	1.5 (0.83)	1.73 (0.8)	** < 0.001**
mMRC score ≥ 2, n (%)	352 (48.2)	93 (63.3)	0.001
Mean BMI (SD)	27.5 (5.5)	26.7 (4.6)	0.173
Median severe exacerbations in the previous year (IQR)	0 (0)	1 (1)	** < 0.001**
≥ 1 severe exacerbations in the previous year, n (%)	0 (0)	45 (3.4)	** < 0.001**
Median BODEx index (IQR)	2 (2)	3 (2)	** < 0.001**
BODEx > 4, n (%)	51 (7)	29 (19.7)	** < 0.001**
Mucus hypersecretion, n (%)	313 (42.9)	81 (55.1)	**0.007**
Long-term home oxygen therapy, n (%)	84 (11.5)	49 (33.3)	** < 0.001**
BiPAP or CPAP, n (%)	40 (6.6)	22 (16.5)	** < 0.001**
Mean no. diseases (SD)	3 (1.86)	2.53 (1.72)	**0.004**
Functional parameters			
FEV_1_/FVC	56.6 (10.7)	54.1 (12.6)	** < 0.001**
FEV_1_ (%)	59.1 (19.0)	51.9 (20.8)	** < 0.001**
FVC (%)	81.9 (21.6)	73.1 (21.8)	** < 0.001**
FEV_1_ ≥ 50%, n (%)	478 (65.5)	71 (48.3)	** < 0.001**
Mean baseline SpO2, % (SD)	95.4 (2.0)	94.7 (2.4)	** < 0.001**
Morbidities, n (%)			
Arterial hypertension	432 (59.2)	90 (61.2)	0.645
Type 2 diabetes mellitus	214 (29.3)	60 (40.8)	**0.006**
Dyslipidemia	403 (55.2)	73 (49.7)	0.218
Obesity	202 (27.7)	37 (25.2)	0.361
Underweight	29 (4)	4 (2.7)	0.361
Ischaemic heart disease	111 (15.2)	40 (27.2)	** < 0.001**
Heart failure	70 (9.6)	24 (16.3)	**0.016**
Atrial fibrillation	117 (16)	35 (23.8)	**0.023**
Heart disease	218 (29.9)	63 (42.9)	0.002
Cerebrovascular accident	50 (6.8)	21 (14.3)	**0.003**
Mood disorder	41 (5.6)	16 (10.9)	**0.018**
Osteoporosis	14 (1.9)	3 (2)	0.921
Bronchial asthma	91 (12.5)	20 (13.6)	0.705
Neoplasia	100 (13.7)	26 (17.7)	0.208
CCI ≥ 3	225 (30.9)	67 (45.6)	**0.001**
CCI, median (IQR)	2 (2)	2 (2)	** < 0.001**

Bold indicates p value < 0.05

*BMI* body mass index; *FEV*_*1*_ forced expiratory volume in 1 s; *FVC* forced vital capacity; *CCI* Charlson comorbidity index score, not age-adjusted; *mMRC* modified Medical Research Council scale; *SpO2* peripheral oxygen saturation by means of pulse oximetry measured with the patient at rest in a sitting position; *BiPAP* bi-level positive airway pressure; *CPAP* continuous positive airway pressure; *IQR* interquartile range

#### According to the GesEPOC 2021 risk groups

Patients designated as high risk patients were slightly older than the low risk patients (69.06 ± 10.22 years vs 66.17 ± 10.53 years; p < 0.001), had a higher smoking load (PYI 48.77 ± 28.34 vs 44.10 ± 25.05; p < 0.001) and a higher prevalence of cardiovascular diseases (Table 3, Additional file 2: Fig. S1).

**Table 3:** Baseline characteristics of patients with chronic obstructive pulmonary disease according to the Spanish guidelines GesEPOC 2021 risk groups

	Low risk patients (n = 258; 29.4%)	High risk patients (n = 619; 70.6%)	p-value
Clinical data			
Mean age, years (SD)	66.2 (10.5)	69.1 (10.2)	** < 0.001**
Female, n (%)	51 (19.8)	125 (20.2)	0.915
Mean pack-year index (SD)	44.1 (25.1)	48.77 (28.3)	**0.023**
Active smoker, n (%)	117 (45.5)	277 (44.7)	**0.032**
mMRC score ≥ 2, n (%)	0 (0)	442 (71.5)	** < 0.001**
Mean BMI (SD)	27.1 (5.09)	27.4 (5.44)	0.587
Median severe exacerbations in the previous year (IQR)	0 (0)	0 (0)	** < 0.001**
≥ 1 severe exacerbations in the previous year, n (%)	0 (0)	147 (23.7)	** < 0.001**
Median BODEx index (IQR)	0 (1)	2 (2)	** < 0.001**
BODEx > 4, n (%)	0 (0)	80 (12.9)	** < 0.001**
Mucus hypersecretion, n (%)	86 (33.5)	308 (49.7)	** < 0.001**
Long-term home oxygen therapy, n (%)	9 (3.5)	124 (20)	** < 0.001**
BiPAP or CPAP, n (%)	3 (1.5)	59 (11.1)	** < 0.001**
Functional parameters			
FEV_1_/FVC	61.1 (7.7)	54.1 (11.6)	** < 0.001**
FEV_1_ (%)	71.2 (15.0)	52.4 (18.4)	** < 0.001**
FVC (%)	91 (21.3)	76.1 (20.6)	** < 0.001**
FEV_1_ ≥ 50%, n (%)	257 (100)	297 (47.9)	** < 0.001**
Mean baseline SpO2, % (SD)	96.1 (1.5)	94.97 (2.2)	** < 0.001**
Morbidities, n (%)			
Arterial hypertension	142 (55.3)	380 (61.3)	0.097
Type 2 diabetes mellitus	51 (19.8)	223 (36)	** < 0.001**
Dyslipidemia	135 (52.5)	341 (55)	0.504
Obesity	63 (24.5)	176 (28.4)	0.241
Underweight	11 (4.3)	22 (3.5)	0.604
Ischaemic heart disease	25 (9.7)	126 (20.3)	** < 0.001**
Heart failure	17 (6.6)	77 (12.4)	**0.011**
Atrial fibrillation	30 (11.7)	122 (19.7)	**0.004**
Heart disease	57 (22.2)	224 (36.1)	** < 0.001**
Cerebrovascular accident	13 (5.1)	58 (9.4)	**0.034**
Mood disorder	14 (5.4)	43 (6.9)	0.416
Osteoporosis	4 (1.6)	13 (2.1)	0.597
Bronchial asthma	29 (11.3)	82 (13.2)	0.431
Neoplasia	34 (13.2)	92 (14.8)	0.536
CCI ≥ 3	56 (21.8)	236 (38.1)	** < 0.001**
CCI, median (IQR)	1 (1)	2 (2)	** < 0.001**

Bold indicates p value < 0.05

*BMI* body mass index; *FEV*_*1*_ forced expiratory volume in 1 s; *FVC* forced vital capacity; *CCI* Charlson comorbidity index score, not age-adjusted; *mMRC* modified Medical Research Council scale; *SpO2* peripheral oxygen saturation by means of pulse oximetry measured with the patient at rest in a sitting position; *BiPAP* bi-level positive airway pressure; *CPAP* continuous positive airway pressure; *IQR* interquartile range

## Discussion

In our study, most of the patients were men between 60 and 80 years of age, mostly overweight, with a moderate to severe degree of airflow obstruction, and heavy tobacco consumption (PYI > 40). Although these characteristics are in line with similar national studies [15], it is particularly striking that up to 45% of the patients were active smokers. This percentage was even observed in highly symptomatic patients. In the Spanish IBERPOC and the ESPIRAL-ES study, 30–55% of the outpatients with COPD were found to continue smoking [16, 17]. The EPOConsul audit of 28 hospitals throughout Spain evaluated the management of patients with COPD through pneumology outpatient follow up and presented a rate of about 31% of active smokers, which is below our regional data [15]. Treatment for smoking cessation is the primary and most cost-effective therapeutic intervention in COPD management [18–20].

Notwithstanding, different studies have revealed that patients with COPD are distinguishable from other smokers insofar as they have a higher degree of dependence and less motivation to quit smoking [21–23]. In addition, tobacco is cheaper in the Canary Islands than on the Spanish mainland and, therefore, more accessible, which could further encourage continued tobacco use in these patients [24]. Moreover, particularly the western Canary Islands have been among the few Spanish regions where tobacco played a major role in their commercial and industrial development. As a result, tobacco use is deep-rooted in the popular culture of the archipelago, which may affect the local perception of its health-threatening effects [25]. The detected high percentage of actively smoking COPD patients in our region shows the need for improved smoking cessation programs.

The patients with COPD on the Canary archipelago exhibit a high prevalence of T2DM and heart disease. T2DM was diagnosed in 30% of our patients. In patients with COPD, T2DM is a common morbidity [26–29], which is related to heart and kidney disease pathogenesis, these latter associated with a higher risk of exacerbation, more symptoms, and poorer survival [30–35]. In the context of cardiovascular disease, the prevalence of cardiac arrhythmia and IHD in the Canary Island population with COPD is approximately 18%. Remarkably, 1 in 3 patients, who were designated high risk according GesEPOC 2021, had some type of heart disease (i.e., cardiac arrhythmia, HF, or IHD). National studies describe a prevalence of cardiac arrhythmia and IHD of about 16% and 12%, respectively [27, 28, 36], which is substantially lower than observed in the Canary archipelago. This data reveals the need to actively search for heart disease in patients with COPD, especially when they belong to the GesEPOC 2021 high-risk group [8].

Of note, patients with COPD who had a history of severe exacerbations exhibited a high prevalence of active smoking, T2DM, and heart disease. A national study performed in 129 hospitals throughout the Spanish territory, with more than 5000 patients included, analysed the characteristics of patients admitted for COPD exacerbation and evidenced a prevalence of active smoking, T2DM, and heart disease of 30, 26, and 30%, respectively, clearly below the prevalence detected in our study (37, 40, 43%, respectively) [37]. All these factors can impact on the clinical progress of these patients in hospital [6, 38, 39]. However, conclusions from our sample should be drawn with caution as there may be a survival bias. Our study population may represent individuals who had recovered from their severe exacerbation for suffering a less severe form of the disease than admitted patients who did not overcome the exacerbation [40]. Hence, larger studies are needed to specifically analyse this group of patients and confirm our results.

This is the first multicentre study in the Canary archipelago that characterises patients with COPD who attend pneumology outpatient services. Its main strength lies in the large sample size and in the good characterization of the population. Although the study was carried out within a specialist-care setting, the high percentage of patients designated low risk according to GesEPOC 2021 may offer an initial view on individuals with relatively mild disease, a point to be confirmed in future studies.

In conclusion, the patient population with COPD in the Canary archipelago is characterised by a high rate of active smokers, T2DM, and cardiovascular disease, which presumably adds complexity to disease management. The high prevalence of heart disease in our patients with COPD, particularly in those exhibiting more symptoms, corroborates the need to actively seek these patients.

## Limitations

The main limitation of this study is a potential information bias due to the recorded variables from the patients' medical records. In addition, we must take into account the limitations of a cross sectional observational study. The temporal sequence of the variables studied could not be established, making it difficult to separate risk factors from prognostic factors. Furthermore, although five of the eight islands of the Canary archipelago are not represented in this study, the selected islands cover more than 80% of the Canary Island population, which adds to the validity of our results offering an overall vision of the patient with COPD in this region.

## Supplementary Information

**Additional file 1: **Table S1. Baseline characteristics of patients with chronic obstructive pulmonary disease according smoking status.

**Additional file 2: **Fig. S1. Morbidity in patients with COPD according to the Spanish guidelines GesEPOC 2021 risk groups.

## Data Availability

The datasets used, analysed or both during the current study are available from the corresponding author on reasonable request. The datasets generated and analysed during the current study are not available to researchers outside of the co-investigators due to data protection laws.

## Abbreviations

AHT: Arterial hypertension
AF: Atrial fibrillation
BMI: Body mass index
BiPAP: Bi-level positive airway pressure
CCI: Charlson comorbidity index score, not age-adjusted
CHF: Chronic heart failure
COPD: Chronic Obstructive Pulmonary disease
CPAP: Continuous positive airway pressure ventilation
CVA: Cerebrovascular accidents
DLP: Dyslipidaemia
FEV1: Forced expiratory volume in 1s
FVC: Forced vital capacity
GesPOC: Spanish Guideline of Chronic Obstructive Pulmonary Disease
GOLD: Global Initiative for Chronic Obstructive Lung Disease
IHD: Ischaemic heart disease
mMRC: Modified Medical Research Council scale
PYI: Pack-year index
SD: Standard deviation
SpO2: Peripheral oxygen saturation by means of pulse oximetry
T2DM: Type 2 diabetes mellitus
