# Supplementary material for: Characteristics of patients referred to Canary Island pneumology outpatient services for chronic obstructive pulmonary disease: the EPOCan study
Source: BMC Res Notes. 2022 Feb 10;15:36. doi: 10.1186/s13104-022-05930-7 (PMC8830167; doi:10.1186/s13104-022-05930-7)
Supplement: Supplementary file 1 — Additional file 1: Table S1. Baseline characteristics of patients with chronic obstructive pulmonary disease according smoking status. [file 13104_2022_5930_MOESM1_ESM.docx]

Table S1 Baseline characteristics of patients with chronic obstructive pulmonary disease according smoking status

|  | **Smoking status** | |  |
| --- | --- | --- | --- |
|  | **Ex- smoker (n = 463; 54%)** | **Active smoker (n = 394; 46%)** | **p-value** |
| **Clinical data** | | | |
| Mean age, years (SD) | 2.5 (1) | 2.3 (1) | **0.003** |
| Female, n (%) | 78 (16.8) | 91 (23.1) | **0.022** |
| Mean pack-year index (SD) | 50.1 (27) | 45.7 (26.5) | **0.018** |
| Mean dyspnoea, mMRC score (SD) | 1.5 (0.8) | 1.6 (0.9) | 0.170 |
| mMRC score ≥ 2, n (%) | 230 (49.7) | 208 (52.8) | 0.363 |
| Mean BMI (SD) | 26.9 (5) | 27.7 (5.6) | **0.036** |
| Median severe exacerbations in the previous year (IQR) | 0 (0) | 0 (0) | **0.030** |
| ≥ 1 severe exacerbations in the previous year, n (%) | 90 (19.4) | 55 (14) | **0.033** |
| Median BODEx index (IQR) | 2 (2) | 2 (2) | 0.349 |
| BODEx > 4, n (%) | 45 (9.7) | 35 (8.9) | 0.684 |
| High risk patients | 334 (72.1) | 277 (70.3) | 0.554 |
| Mucus hypersecretion, n (%) | 207 (44.7) | 183 (46.4) | 0.611 |
| Long-term home oxygen therapy, n (%) | 83 (17.9) | 48 (12.2) | **0.020** |
| BiPAP or CPAP, n (%) | 39 (10.1) | 22 (6.5) | 0.086 |
| Mean no. diseases (SD) | 2.6 (1.8) | 2.6 (1.8) | 0.903 |
| **Functional parameters** | | | |
| FEV_1_/FVC | 57 (10.1) | 55.1 (12) | **0.014** |
| FEV_1_ (%) | 57.1 (19.4) | 58.7 (19,3) | 0.225 |
| FVC (%) | 78.4 (23.1) | 83.02 (19.9) | **0.002** |
| FEV_1_ ≥ 50%, n (%) | 283 (61.1) | 254 (64.5) | 0.313 |
| Mean baseline SpO2, % (SD) | 95.27 (2.0) | 95.29 (2.1) | 0.879 |
| **Morbidities, n (%)** | | | |
| Arterial hypertension | 271 (58.5) | 242 (61.4) | 0.390 |
| Type 2 diabetes mellitus | 162 (35.0) | 109 (27.7) | **0.022** |
| Dyslipidemia | 239 (51.6) | 227 (57.6) | 0.079 |
| Obesity | 104 (22.5) | 126 (32.1) | **0.002** |
| Underweight | 16 (3.5) | 15 (3.8) | 0.778 |
| Ischaemic heart disease | 91 (19.7) | 56 (14.2) | **0.035** |
| Heart failure | 58 (12.5) | 34 (8.6) | 0.066 |
| Atrial fibrillation | 91 (19.7) | 59 (15.0) | 0.072 |
| Heart disease | 166 (35.9) | 108 (27.4) | **0.008** |
| Cerebrovascular accident | 46 (9.9) | 25 (6.3) | 0.057 |
| Mood disorder | 23 (5.0) | 32 (8.1) | 0.060 |
| Osteoporosis | 9 (1.9) | 8 (2.0) | 0.928 |
| Bronchial asthma | 49 (10.6) | 60 (15.2) | **0.042** |
| Neoplasia | 73 (15.8) | 51 (12.9) | 0.242 |
| CCI ≥ 3 | 175 (37.9) | 114 (28.9) | **0.006** |
| CCI, median (IQR) | 2 (2) | 2 (2) | **0.008** |
| *BMI* body mass index; *FEV_1_* forced expiratory volume in 1 second; *FVC* forced vital capacity; *CCI* Charlson comorbidity index score, not age-adjusted; *mMRC* modified Medical Research Council scale; *SpO2* peripheral oxygen saturation by means of pulse oximetry measured with the patient at rest in a sitting position; *BiPAP* bi-level positive airway pressure; *CPAP* continuous positive airway pressure; *IQR* interquartile range. | | | |
