# Supplementary material for: Characteristics of patients referred to Canary Island pneumology outpatient services for chronic obstructive pulmonary disease: the EPOCan study
Source: BMC Res Notes. 2022 Feb 10;15:36. doi: 10.1186/s13104-022-05930-7 (PMC8830167; doi:10.1186/s13104-022-05930-7)
Supplement: Supplementary file 2 — Additional file 2: Fig. S1. Morbidity in patients with COPD according to the Spanish guidelines GesEPOC 2021 risk groups. [file 13104_2022_5930_MOESM2_ESM.docx]

**Fig. S1** Morbidity in patients with COPD according to the Spanish guidelines GesEPOC 2021 risk groups


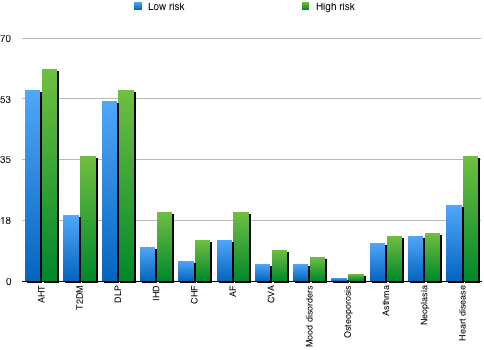


Abbreviations: AHT: arterial hypertension; AF: atrial fibrilation; CHF: chronic heart failure; CVA: cerebrovascular accidents; DLP: dyslipidemia; IHD: ischeamic heart disease; T2DM: type 2 diabetes mellitus
